# Performance and Reliability Analysis of Water Distribution Systems under Cascading Failures and the Identification of Crucial Pipes

**DOI:** 10.1371/journal.pone.0088445

**Published:** 2014-02-13

**Authors:** Qing Shuang, Mingyuan Zhang, Yongbo Yuan

**Affiliations:** Department of Construction Management, Dalian University of Technology, Dalian, Liaoning, China; Plymouth University, United Kingdom

## Abstract

As a mean of supplying water, Water distribution system (WDS) is one of the most important complex infrastructures. The stability and reliability are critical for urban activities. WDSs can be characterized by networks of multiple nodes (e.g. reservoirs and junctions) and interconnected by physical links (e.g. pipes). Instead of analyzing highest failure rate or highest betweenness, reliability of WDS is evaluated by introducing hydraulic analysis and cascading failures (conductive failure pattern) from complex network. The crucial pipes are identified eventually. The proposed methodology is illustrated by an example. The results show that the demand multiplier has a great influence on the peak of reliability and the persistent time of the cascading failures in its propagation in WDS. The time period when the system has the highest reliability is when the demand multiplier is less than 1. There is a threshold of tolerance parameter exists. When the tolerance parameter is less than the threshold, the time period with the highest system reliability does not meet minimum value of demand multiplier. The results indicate that the system reliability should be evaluated with the properties of WDS and the characteristics of cascading failures, so as to improve its ability of resisting disasters.

## Introduction

The stability and reliability of Water distribution systems (WDSs) is one of the important factors in ensuring public safety and the continuous operation of urban functions. Such functions include water supply, infrastructure construction and industrial development, etc. It is also the key field for infrastructure construction. The WDS is a large scale network system with complex topological structure [1]. Its functions are designed to convey volumes of water to customers under adequate pressure. Nowadays, along with the increased population and population density, WDS is developing into wide-range supply which carries fluid under high or less pressure. A WDS can be represented as a spatially networks of multiple interconnected components. Pipes can be represented as links. Junctions, reservoirs and consumers can be represented as a collection of nodes. With the link-node representation of physical components in WDS, complex network analysis can be applied to evaluate the system reliability.

Complex networks are an essential part in the understanding of many natural systems [2]. A complex network is a network with non-trivial topological features, which often occur in real life. Complex networks analysis provides a way to understand the meaning and functions of the system [3]. It focuses on predict the networked system behavior on the basis of measured structure. Albert et al. [4] have found that the scale-free networks have strong robustness under random disturbance, but it is very vulnerable under intentional attacks. These important discoveries have made the network security under abnormal conditions become a hot issue in this field. Cascading failures is a conductive failure process in the field of network security [5]. When the network encounters natural or man-made disasters, i.e. network attacks and random failures, the minor anomalous event of a point may spread to the whole system through cascade reaction, leading to large-scale consequences and secondary failures. Many models have been provided to investigate the cascading failures. The present studies mainly focus on: (1) the network reliability and topology structure after remove some nodes or links [6]–[8]; (2) the formation conditions and reasons of cascading phenomena and the network dynamics in networks or weighted networks [9]–[10]; and (3) the metrics of network robustness and the sequent network optimization and design [11].

For the real-world networks, the cascading failures of infrastructure systems have been proposed as well. The power grids of North America and the Western United States are two key studies in this field [12]–[15]. Besides, the Internet network [16], the power grids of European [17]–[18] and Italian [19], and other kinds of power systems [20] and traffic networks [21] are also the focus of studies. In these studies, most cascading failures use the virtual network simulation method which measures the network load by the topological property, such as the betweenness and degree. The betweenness is defined as the total number of the shortest paths that pass through the vertex [22]. The degree is defined as the number of edges connected to the vertex [23]. Therefore betweenness and degree basically measure the topological structure of a network. This method fits for the disaster simulation under uncertainties and the rapid assessment on disasters. However, it ignores the properties of city lifeline system as an entity network. Using the betweenness or degree to represent the actual network flow cannot guarantee the accuracy of the calculation results. Therefore, the results cannot be directly applied to the decision-making.

System reliability is defined as the ability of the system to complete the scheduled functions in a certain period under the given working state [24]–[25]. There are two failure mechanisms of a WDS [26]: mechanical and hydraulic failures. Mechanical reliability is defined as the probability that the WDS and its components are operational. Mechanical reliability focuses on a topological perspective. Hydraulic reliability is defined as the probability that the WDS meet flow and pressure requirements. Hydraulic reliability considers failures in meeting consumer demand. The reliability in this paper combined these two types of reliability. With the topological dynamics changing, the scheduled functions refer to ensuring the water demand and hydraulic pressure required in the daily life of customs in normal conditions; under failure conditions, the water supply and hydraulic pressure would not be lower or higher than the specified limit.

The purpose of this paper is to study the propagation characteristics of cascading failures in the WDS and put forward the methods to identify the crucial pipes. The methods from complex networks and engineering are adopted. The simulation of the cascading failures in WDS is required to meet the equilibrium of water supply and demand. The main task is adopting the numerical simulation technology to depict the damage process of WDS in cascading failures. The network dynamics are used with failure propagation. The uncertain factors, i.e., the nodal head bounds, daily demand multipliers and the water demand have been taken into consideration. The crucial pipe is identified by its vulnerability and sensitivity to cascading failures. By identifying the most crucial pipes, one can effectively protect the network to avoid cascading failures and build attack-robust networks.

## Methods

### 1. Reliability Assessment

The reliability is defined as the probability that the WDS meet flow and pressure requirements under the possible mechanical failure scenarios (e.g., pipe breaks). The definition of system reliability given by Zhuang et al. [27] is adopted. Mathematically, the reliability can be expressed as the ratio of the available flow to the require demand. The condition includes normal condition and failure condition. The reliability of node and system is expressed as:
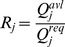
(5)

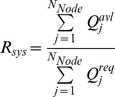
(6)where *R_j_* is the *j*th node reliability; *R_sys_* is the reliability of WDS; *N_Node_* is the number of nodes; *Q_j_^avl^* is the available flow at *j*th node (L/s); *Q_j_^req^* is the require demand when *j*th node is under normal condition.

### 2. WDS Topological Structure Expression

Before analogue simulation and calculation, the first step is to input the graphic information of the system into the computer and set up the model. A WDS can be analyzed by the methods of graph theory based on its topology structure. Graph theory is the study of graphs. A graph consists of a set of nodes and links, representing the interactions among them. A graph is customarily depicted the nature of the links between nodes. A directed graph is one in which links have orientation [28]. A WDN is a directed graph due to the operational flow and pressure requirements. The reservoirs, junctions and customers are described as nodes; while the pipes, pumps and valves are represented as links. The adjacent nodes in the graph are connected by the links in most of the cases.

The matrix is used as an effective tool to depict the properties of the graph in network modeling. The adjacency matrix and incidence matrix are the most common ones. The adjacency matrix *A* is used to represent the relationship between the nodes in the network. The values for the element *a_ij_* are: 0 and 1. When the node *i* and node *j* is connected by a pipe, the value is 1; when the node *i* and node *j* is unconnected, the value is 0.
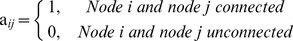
(1)


Incidence matrix *N* is for describing the relationship between nodes and pipes. The row represents the nodes and the column represents the pipes, respectively. In the network graph, each node and pipe is numbered by consecutive number from 1. The information of node *i* is recorded in the *i*th row and that of pipe *j* is recorded in the *j*th column. The element *n_ij_* is expressed as:
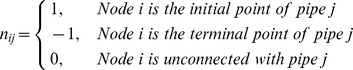
(2)


### 3. Modeling of WDS Based on Cascading Failures

#### 3.1 The Load of WDS Nodes

In view of the actual physical meaning of WDS, we adopt the nodal pressure head as the load. The load of nodes is a relevant quantity, which can be material, information and energy [5], and can be concrete or abstract. With the passage of time, the load of the nodes exchanges along the connected edge between each of the node pair. The WDS is a kind of material loading network. It distributes water from the reservoirs to the customers. In order to meet the customers' daily needs, each component of the WDS must be able to provide required water demand and pressure head under both normal and failure conditions. Therefore, we define the initial load of the node as the service head *H_s_*. The service head ensures that all the imposed demands can be satisfied.

#### 3.2 The Relationship between Nodal Capacity and Initial Load

The nodal capacity is the maximum load the node can bear. In the place of residence or business, the nodal pressure should not be too high or too low. This because the low pressure leads to flow reductions or blanking; high pressure causes the pipe leakage and even the burst of ageing pipes so that losing its service functions. In general, for each node, there are three kinds of pressure head:

1. *H_min_*, the acceptable minimum level of pressure. If the nodal pressure head is lower than *H_min_*, the node loses its service function. Therefore, the minimum capacity is defined as the nodal minimum head.
2. *H_s_*, the service level of pressure to meet all the imposed demands. Only when each nodal pressure head is higher than service head, the WDS can be performing normally;
3. *H_max_*, the acceptable maximum level of pressure that a node can bear. Then, define the maximum capacity of the node. In a man-made system, the nodal capacity is severely limited by cost. For WDS, the pressure head, *H*, at each demand node is always within a specified range of a minimum head *H_min_* and a maximum *H_max_* [29] when it's in normal operating conditions. Besides, there is a direct relationship between pipe leakage and service pressure. The water leakage increases with pressure [30]. In order to avoid the leakage of ageing pipes caused by over-high pressure, the maximum capacity of the node should be defined. Suppose *α* is the tolerance parameter. It is possible to assume that the maximum capacity is proportional to the initial load *H_s_*. The maximum capacity can be expressed as:
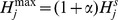
(3)where *H_j_^max^* is the *j*th nodal maximum head capacity; *H_j_^s^* is the *j*th nodal service head. *α* allows a systematic evaluation of the aggregated performance of water distribution network element during cascading propagation [31]. The bigger *α* is, the higher the capacity constraint will be, which means less likely the node would fail.

To represent actual flows supplied to customers under abnormal condition, the available nodal demand is expressed as a function of nodal pressure head. Considering the formulation proposed by Wagner et al. [32], and take the maximum head capacity into account, the function can be expressed as follows:
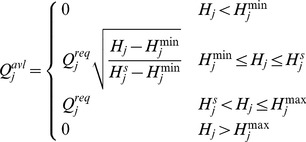
(4)where *Q_j_^avl^* is the flow delivered to the *j*th node (L/s); *Q_j_^req^* is the require demand when *j*th node is under normal condition. *H_j_*, *H_j_^min^*, *H_j_^max^* and *H_j_^s^* represent the calculated head, the minimum head, the maximum head and the service head at *j*th node, respectively.

For the above three kinds of pressure heads, there are four states of water supply: (1) nodes are completely shut off when the pressure head is lower than the minimum head; (2) the customers' demands is supplied at a reduction level when the pressure head is higher than the minimum head and lower than the service head; (3) nodes meet the customers' demand when the pressure is higher than the service head; and (4) nodes are closed when the pressure head is higher than the maximum head.

#### 3.3 The Cascading Dynamics

The load on the network is in dynamic changes, especially when the network structure transforms. For example, the load is redistributed due to some nodes or pipes shut off. In general, the network node and edge have a limited bearing capacity. If the maximum load (capacity) is exceeded, the network equilibrium will be broken and the load will be redistributed.

**Figure 1:**
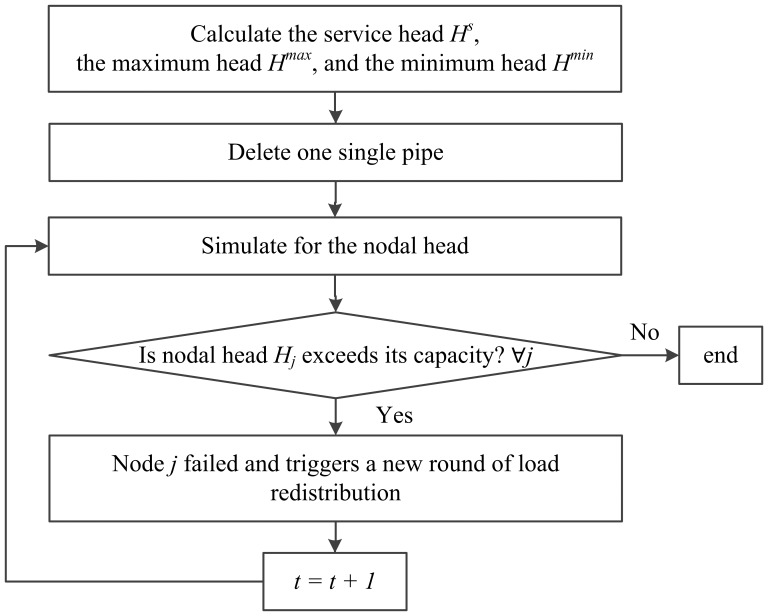
Iterative process of the cascading dynamics of WDS.

When a pipe of the WDS is closed for failure condition, it is equivalent to removing an edge of the network. Then it triggers the network flow to be redistributed among all nodes. The artificial time step *t* (*t = 1, 2, …*) is introduced to monitor the process of cascading failures. At time *t*, if the nodal pressure head *H_j_* exceeds its capacity (above the maximum head or below the minimum head), this node fails to provide required water. The failure triggers the reduction of its downstream pipes. The WDS pressure is a spatial vector and the pressure on each node is interdependent. The pressure of one node changes leads to other node pressure changes to varying degrees. In this situation, a new round of load redistribution occurs and leads to cascading failures. The iterative process continues until there are no failure nodes or pipes produced, which implies the cascading can be considered stopped. The iterative process is described in Figure 1.

In the operation of WDS, two or more components come to failure together rarely happens [33]. Therefore, this paper only considers the situation of single-pipe failure. Note that this model can be easily extended to multiple-pipe failure.

### 4. Hydraulic Simulation

The EPANET [34] simulation engine has been used for the WDS hydraulic simulation. EPANET is a water distribution system modeling software developed by the United States Environmental Protection Agency's (EPA). EPANET is available as an open-source toolkit. Its Programmer's Toolkit is a dynamic link library (DLL) of functions that allow developers to customize EPANET to their own needs.

In actual operation, the network distributes water along the pipelines. The water demands of the customers are time-varying and uncertain. Therefore, the water demand multipliers need to be considered as an uncertain and dynamic variable. To evaluate the system reliability with the time-varying demands, a water demand pattern is established by the extended period hydraulic simulations module of EPANET. The time step is set as one hour. Use MATLAB 2010a to implement modeling of different stages and the EPANET Toolkits' functions.

### 5. Assumptions and Algorithm Flowchart

Assumptions:

1. The pipes of the WDS have just two states: operation or failure. Demand nodes have three states: operation, failure or intermediate state. The intermediate state means the water demand at the node is partly met. The amount of water flow is available but less than the require demand.
2. A pipe is operational if the water can flow smoothly from its initial node to its terminal node.

3a. A node is operational if its service function is effective, i.e., the pressure head of the node is no less than the minimum head and no higher than the maximum capacity.
3b. When a node is in failure, its downstream connected edge is in failure also.
3c. If the upstream pipes of a node are all in failure, the node is failed as an unintended isolated node due to it has no source of water supply.

For a WDS, three kinds of failure states are existed based on the assumptions. (1) The pressure head at a node exceeds its maximum capacity; (2) The pressure head at a node is lower than its minimum capacity; and (3) A node is unintended isolated as a result of all its upstream pipes are in failure state.

**Figure 2:**
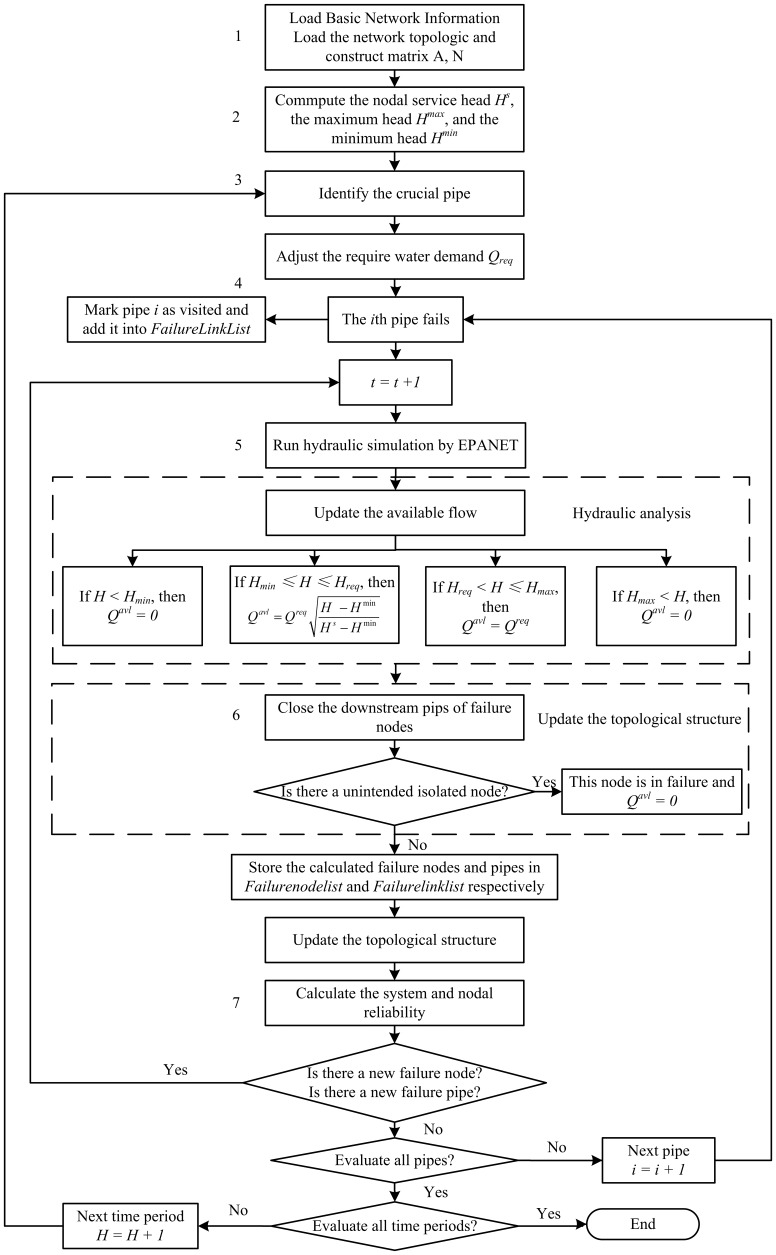
Simulation flowchart of pipe failure under WDS cascading failures.

According to the simulation flow of cascading failures, the algorithm flowchart is described as follows (Fig. 2):

1. Input the basic information of a WDS, including diameters, lengths and roughness coefficients of pipes, nodal elevations, base demands, and demand multipliers. Load the network topologic and construct the adjacency matrices *A* and the incidence matrices *N*.
2. Calculate the initial load (service pressure head) and its maximum and minimum capacity.
3. Adjust the require water demand of customers according to the demand multipliers. Set the initial time step as *t = 1*. *t = 1* indicates that each node of a WDS meet its pressure head. *t = 2* describes the hydraulic state after a certain pipe failed. *t≥3* represents each stage of the cascading failures where new nodes or pipes fail as a result of flow redistribution
4. Assume each of the pipes is in failure successively. Define FailureNodeList and FailureLinkList as the set of failed nodes and failed pipes, respectively. Set FailureNodeList = {} and FailureLinkList = {}. If there is a subsequent failure, the node or pipe index is appended to the FailureNodeList and FailureLinkList, respectively. These two sets are used to update the system topological structure.
5. Run hydraulic analysis in failure conditions. Simulate the running state of the water distribution network by EPANET and obtain the pressure head on each node. A node is recognized as a subsequent failure node if its load exceeds the range of capacity. Update the available flow according to Eq. (4).
6. Update the topological structure under failure conditions. Close the downstream pipes of failure nodes. Judge whether there is unintended isolated node; if so, this node is in failure. Store the calculated failure nodes and pipes in FailureNodeList and FailureLinkList respectively and update the topological structure of the WDS.
7. Calculate the system and nodal reliability. Repeat steps (3)–(6) until the failure of all the pipes have been simulated. Use Eq. (5) and (6) to calculate the reliability of each node and the whole network, respectively.

## Results and Discussion

**Figure 3:**
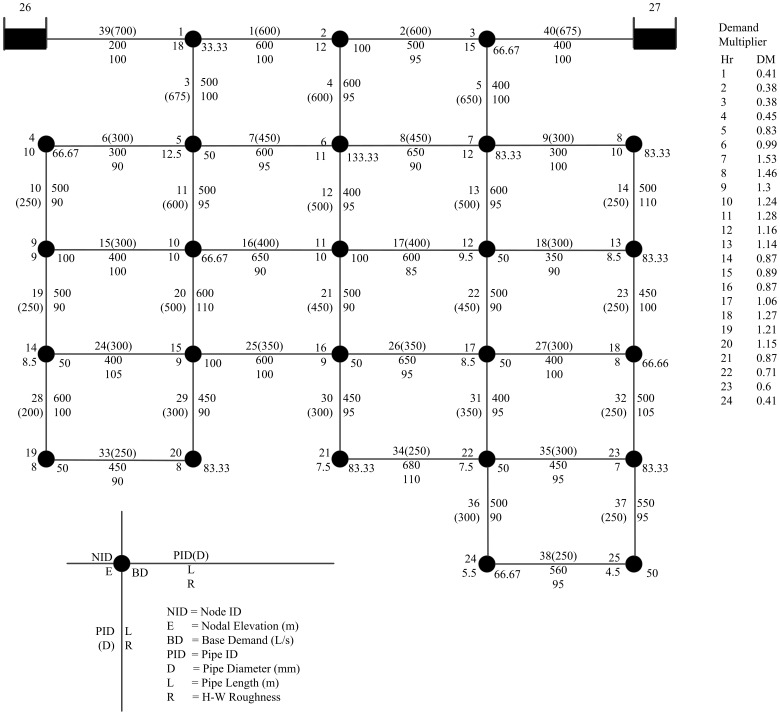
The layout of the example WDS.

The proposed methodology is applied to a WDS from Islam et al. [35]–[36]. The network includes two reservoirs, twenty-five water demand nodes and forty pipes. The topological structure, nodal elevation, base demand, pipe diameter and length as well as other basic information of the network has been shown in Figure 3. The total length of all pipes is 19.5 km. The water is supplied by gravity from the elevated reservoirs (reservoir 26 and reservoir 27) with the total heads of 90 m and 85 m, respectively. The pipe lengths range from 100 m to 680 m and the diameters vary from 200 mm to 700 mm. The basic demand is in the range of 33.33 l/s to 133.33 l/s. The demand multipliers (DMs) are considered as ranging from 0.38 at 2am to 1.53 at 7am. The Hazen-Williams formula is used to calculate the head loss.

As the original literature has not given the minimum head, we use the EPANET to solve hydraulic calculation on the network under normal condition (no pipe failure occurred). The pressure head at each node is shown in Table 1. We can find from the results that the pressure heads of node 24 and 25 are lower than 65 m for their long distance away from the reservoirs. Considering the minimum head have a certain tolerance, here assume *H_min_* = 50 m. The maximum head can be calculated by Eq. (1)

**Table 1:** Pressure heads of each node under the normal condition.

Node ID	Pressure Head (m)	Node ID	Pressure Head (m)	Node ID	Pressure Head (m)
1	86.91	10	79.30	19	69.65
2	84.30	11	78.88	20	70.27
3	84.35	12	77.89	21	69.91
4	78.66	13	74.54	22	68.82
5	82.92	14	74.91	23	66.85
6	82.14	15	76.20	24	64.70
7	82.16	16	75.88	25	64.36
8	77.09	17	74.24		
9	76.14	18	71.53		

### 1. Failure rate and edge betweenness

**Figure 4:**
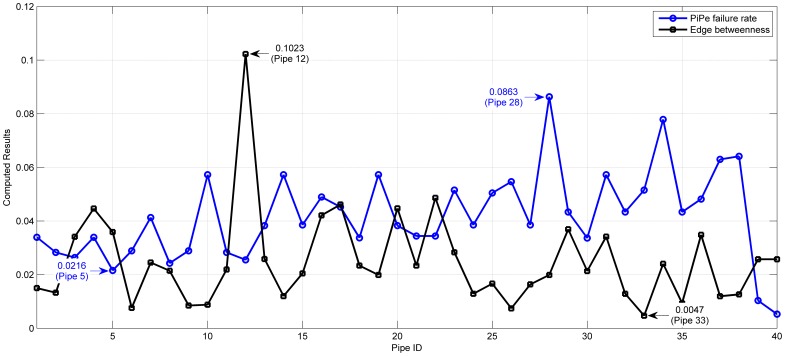
Failure rate and edge betweenness of WDS.

Figure 4 shows the failure rate of each pipe and the edge betweenness of pipe network. The failure rate per year per unit length of pipe is calculated by the function proposed by Su and Mays [37].

Calculate the edge betweenness of the network by Matlab BGL toolbox, and then normalize the results. It is obvious that the pipe 39 and 40 which are directly connected with the reservoirs are the most crucial pipes. The failures of these two pipes directly lead to the failure of the downstream water demand nodes. Therefore, in the study of pipes, it is necessary to research the reliability of pipes except pipe 39 and 40. Figure 4 shows pipe failure rate and the edge betweenness. It is visible that except pipe 39 and 40, the minimum pipe failure rate is 0.0216 (pipe 5) and the maximum one is 0.0863 (pipe 28). So, it is difficult to distinguish the crucial pipe. From the edge betweenness, we can see that the top three maximums are 0.1023 (pipe 12), 0.0486 (pipe 22) and 0.0461 (pipe17), and the minimum is 0.0047 (pipe 33).

### 2. Peak and Period of Cascading Failures in in WDS

**Figure 5:**
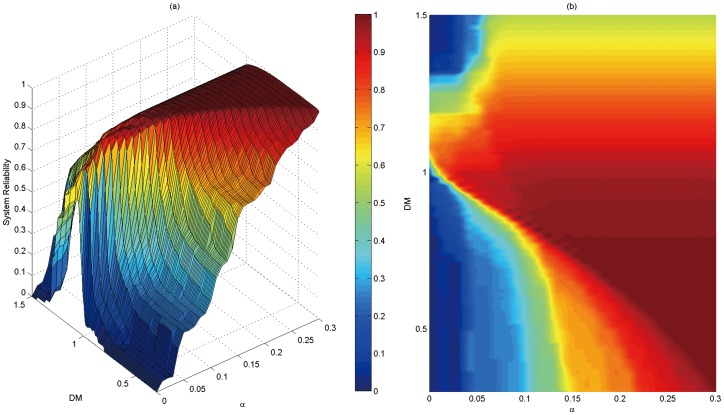
Variation diagram of WDS reliability with *DM* and *α*.

The value of *α* measures the limit of capacity caused by cost factors in the initial stage of WDS construction, or that caused by ageing and corrosion in the operation stage of WDS. Despite the simple meaning of *α*, it provides the method to evaluate the overall performance of the system in the cascading failures. Figure 5 shows the reliability of the system when *DM* value is in the range of 0.3∼1.5 and *α* is ranging from 0 to 0.3. Figure 5 (a) presents the 3D view, (b) is an overhead view. Suppose a certain pipe fails, the simulation is carried out. The system reliability is calculated for the whole system if no subsequent failures are found. Simulate the 38 pipes successively. The system reliability in Figure 5(a) is the average reliability of these 38 pipes. The simulation result shows that, (1) the larger *α* is, the better the invulnerability of WDS against cascading failures will be. This is because under the given load redistribution strategies, the higher capacity of network design means a stronger potential ability of the system in assimilating and accommodating local failure and a better ability of the network to deal with of cascading failure. (2) as the computational condition of *H_max_*, *α* enlarges the node pressure from maximum 86.91 m to 112.98 m, the relative amplification is 30%. With the increase of *α*, the system reliability increases in the whole with a remarkable change from 0 to 1.0 and the relative growth is 100%, 70% higher than the maximum pressure head. In the limited capacity of the node, the small disturbance of WDS has a significant effect on water supply.

Analysis from the perspective of *DM* shows that *DM* has a great effect on the propagation of cascading failures in WDS. The simulation results shows, (1) when *DM>1*, system reliability is low after coming to the stable state and the stable value becomes smaller with the increase of *DM*. For example, when *DM = 1.3*, the stable value of system reliability is 0.7179; *DM = 1.5*, the stable value of system reliability is 0.5557; when *DM<1*, the stable value of system reliability is much higher. For example, when *DM = 0.5*, the stable value of system reliability is 0.9993. (2) when *α* is given, there exists a threshold *DM_c_* of *DM* making the system reliability always reach a peak over a period of time. When *DM* is at its threshold *DM_c_*, the system reliability levels off to *DM_c_* and rises rapidly. After reaching the peak, it decreases rapidly. Moreover, *DM_c_* decreases with the increase of *α*. For example, when *α = 0* and *DM = 1.08*, the peak is *Rsys = 0.7161*; when *α = 0.3* and *DM = 0.33*, *Rsys = 1.0*. (3) When *DM<1*, as DM decreases, the system reliability needs more time to be stable and this time gets longer with the decrease of *DM*. For example, when *DM = 0.7*, the system reliability gets stable at *α = 0.18*; when *DM = 0.3*, the system reliability has not yet reached a stable value at *α = 0.3*. Therefore, in the simulation of the pipe network, the *DM* should be considered to make the evaluation result more accurate.

### 3. Identification of Crucial Pipe

The WDS simulation is carried out based on the flowchart in Figure 2. *α* is set in six types from 0.05 to 0.3. Steps 3–7 in Figure 2 are carried out after setting *α* and *DM* to a certain value. The subsequent failures are considered. If no new failures are found, the cascading failure stops and the system remain stable. The system reliability can be obtained when the system come to stable state again. The 38 pipes are simulated successively. The lowest system reliability of this period and its related pipe ID are recorded in Table 2.

**Table 2:** Crucial pipes ID (CPID) under six types of *α* and their corresponding system reliability *R_sys_*.

		*α = 0.3*		*α* = 0.25		*α* = 0.2		*α* = 0.15		*α* = 0.1		*α* = 0.05	
DM	Hour	CPID	*R_sys_*	CPID	*R_sys_*	CPID	*R_sys_*	CPID	*R_sys_*	CPID	*R_sys_*	CPID	*R_sys_*
0.41	1	32	0.9956	32	0.8861	1	0.7407	2	0.6482	2	0.2593	5	0.1111
0.38	2	32	0.9993	32	0.8885	1	0.7407	5	0.6019	2	0.2593	5	0.1111
0.38	3	32	0.9993	32	0.8885	1	0.7407	5	0.6019	2	0.2593	5	0.1111
0.45	4	32	0.9881	32	0.9233	1	0.7407	5	0.6759	2	0.2593	5	0.1111
0.83	5	3	0.9151	3	0.9151	3	0.9151	3	0.9151	32	0.463	2	0.1574
0.99	6	3	0.7651	3	0.7651	3	0.7651	3	0.7651	30	0.75	30	0.2315
1.53	7	3	0.1961	3	0.1961	3	0.1961	3	0.1961	3	0.1961	3	0.1961
1.46	8	3	0.2285	3	0.2285	3	0.2285	3	0.2285	3	0.2285	3	0.2285
1.3	9	3	0.3181	3	0.3181	3	0.3181	3	0.3181	3	0.3181	5	0.2315
1.24	10	3	0.3793	3	0.3793	3	0.3793	3	0.3793	3	0.3793	9	0.2315
1.28	11	3	0.3336	3	0.3336	3	0.3336	3	0.3336	3	0.3336	5	0.2315
1.16	12	3	0.4921	3	0.4921	3	0.4921	3	0.4921	3	0.4921	9	0.2315
1.14	13	3	0.5214	3	0.5214	3	0.5214	3	0.5214	3	0.5214	11	0.2315
0.87	14	3	0.8833	3	0.8833	3	0.8833	3	0.8833	36	0.463	2	0.1574
0.89	15	3	0.8662	3	0.8662	3	0.8662	3	0.8662	30	0.75	8	0.1574
0.87	16	3	0.8833	3	0.8833	3	0.8833	3	0.8833	36	0.463	2	0.1574
1.06	17	3	0.6649	3	0.6649	3	0.6649	3	0.6649	3	0.6649	22	0.2315
1.27	18	3	0.3465	3	0.3465	3	0.3465	3	0.3465	3	0.3465	5	0.2315
1.21	19	3	0.4299	3	0.4299	3	0.4299	3	0.4299	3	0.4299	5	0.2315
1.15	20	3	0.508	3	0.508	3	0.508	3	0.508	3	0.508	9	0.2315
0.87	21	3	0.8833	3	0.8833	3	0.8833	3	0.8833	36	0.463	2	0.1574
0.71	22	32	0.9259	32	0.9259	32	0.9259	2	0.7037	5	0.3148	5	0.1111
0.6	23	32	0.9305	32	0.9305	32	0.787	1	0.7037	5	0.2593	5	0.1111
0.41	24	32	0.9956	32	0.8861	1	0.7407	2	0.6482	2	0.2593	5	0.1111

The simulation results are shown in Table 2. The first column is the *DM* per hour; the second column is hours; the column 3, 5, 7, 9, 11, and 13 list the ID of crucial pipes under different tolerance parameters *α* and their corresponding system reliability *R_sys_*, respectively.

**Figure 6:**
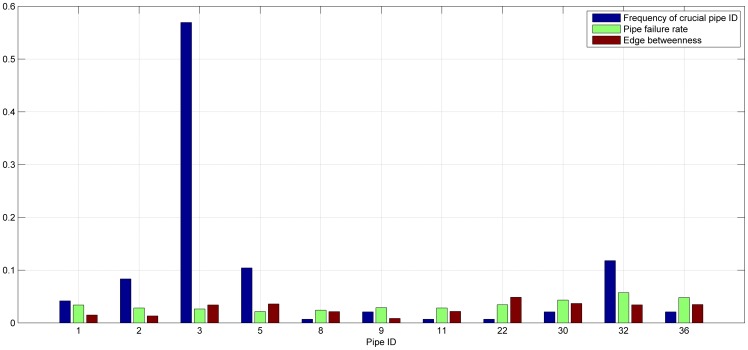
Frequency diagram of crucial pipes.

It can be found in Table 2 that the vulnerable pipes ID are 1, 2, 3, 5, 8, 9, 11, 22, 30, 32, and 36. Among them, in order to find which one is the most crucial pipe, we calculate the frequency of each pipe ID which appears in Table 2. It is done by calculating the sum of each pipe ID over sum of all pipes ID. The comparison of the frequency of crucial pipes, the failure rate and the edge betweenness is shown in Figure 6. We can see that pipe 3 is the most crucial pipes because its frequency is significantly higher than that of other pipes. The distribution period of pipe 3 covers 5–21, the corresponding DM≥0.83 and the maximum value reaches 1.53. In addition to pipe 3, the other crucial pipes are pipe 32, 5, 2, 1, (9, 30, 36) (figures in brackets denote the equally important). Besides, from the geographical distribution of pipes, pipe 1, 2, 3, 5 are relatively closer to the reservoirs while pipe 30, 32, 36 are relatively far away from the reservoirs.

Compare the pipe failure rate and edge betweenness shown in Figure 4, we can see that the crucial pipes 3, 32, 5, 2, and 1 got from the simulation of cascading failure are not the maximum values in the failure rate or the edge betweenness. On the contrary, pipe 2, 3, 5 have a relative small failure rate which lead to serious network avalanche. On the other hand, betweenness used as a way to measure network characteristics has been widely seen as the index of the importance. The existing studies on the robustness of the lifeline system have taken the edge or node betweenness as network initial load [12], [14], [38], based on what the further analysis on network characteristics and emergency strategies have been proposed. The simulation results show that the failure of pipe 12 with the largest betweenness does not cause a wide range of network avalanche. Using betweenness or degree instead of lifeline network entity flow is a method that analyses different kinds of lifeline networks (WDS, transportation, communication, power grid, etc.) in the same way. It ignores the characteristics of the network flow, service function and constraints. The results cannot be directly applied to adjusting the lifeline network relationship or adjusting the network flow.

### 4. Discussion of the Most Reliable Time Period

Except the multiple identical minimum values occurred at *α* = 0.05, the minimum value under other conditions are within the time period of *H* = 7 period (*R_sys_* = 0.1961). The *DM = 1.53* at 7am is the largest water demand during the day. It can be verified that the peak of water usage is the most vulnerable period of a WDS. System reliability decreases with the increase of *DM*.

Table 3 presents the maximum value (relative maximum) of the minimum values of system reliability in one day. The value is selected by the maximum value of system reliability from each column in Table 2. The time period 2 and 3 has the minimum *DM* (*DM = 0.38*). Except for the situation of *α* = 0.3, the relative maximum values of system reliability do not meet the minimum *DM*, but 0.41, 0.6, 0.71, 0.87, 0.99 and 1.21, respectively.

**Table 3:** The relative maximum of the minimum values of system reliability under six states of *α*.

*α* = 0.3		*α* = 0.25		*α* = 0.2	
Hour (DM)	*R_sys_*	Hour (DM)	*R_sys_*	Hour (DM)	*R_sys_*
2 (0.38), 3 (0.38)	0.9993	23 (0.6)	0.9305	22 (0.71)	0.9259

*α* = 0.15		*α* = 0.1		*α* = 0.05	
Hour (DM)	*R_sys_*	Hour (DM)	*R_sys_*	Hour (DM)	*R_sys_*
5 (0.83)	0.9151	6 (0.99)	0.75	6 (0.99)	0.2315

**Figure 7:**
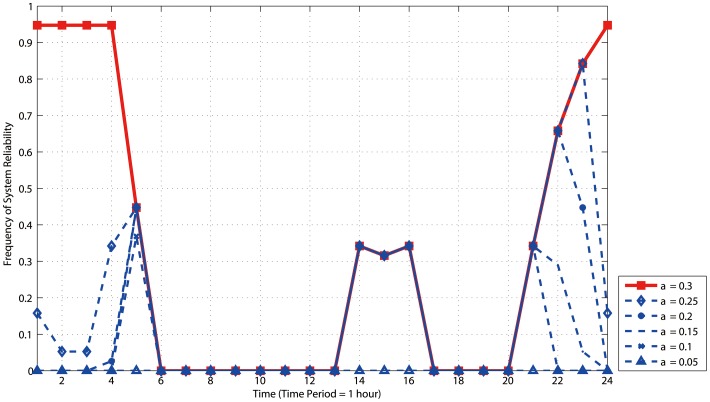
Frequency diagram of the maximum value (*R_sys_* = 1) of system reliability within 24 hours.

In order to make further analysis on the most reliable period in a day, Figure 7 censuses the frequency of *R_sys_* = 1 in each period. In order to avoid the deviation of results, the frequency of *R_sys_* = 1 when *α*>0.3 is calculated and analyzed. The statistical results show that the frequency of *R_sys_* = 1 does not change with *α* increased when *α*≥0.3. Hence, in this example, there is a threshold of tolerance parameter *α_c_* = 0.3. When *α*>*α_c_*, the period with the highest system reliability meets the time period with *DM*<1; when *α*<*α_c_*, the period with the highest system reliability does not meet the minimum *DM*. Therefore, when select the repair period of a WDS, one cannot simply choose the period with the minimum *DM*, but choose the period with the highest system reliability based on the operation status. What's more, the crucial pipes apt to causing large-scale cascading failure should be avoided also.

## Conclusions

The cascading dynamics of WDS in failure condition and the identification of crucial pipes have been discussed in this paper. The propagation of cascading failures in WDS is measured by the damage of certain pipe. The identify factor of crucial pipes is the system reliability after the network restores to stable state. The cascading failure simulation of WDS has taken the nodal pressure head, available water flow, daily demand multipliers and the topological structure in to account. Based on this method, using MATLAB to call EPANET source program is realized. The case study has demonstrated the applicability of this method. The results verified that this method is suitable for WDS and can effectively identify the crucial pipes.

In the network cascading dynamics modeling, it is generally assumed that once the load of a node or edge in the network exceeds its maximum capacity, the corresponding node or edge is avalanched and out of function, triggering the redistribution of network load and cascading failures. However, in the real-world network, there is always some kind of emergency mechanism and emergency response. When a failure occurs, the external emergency power can be involved in to exert its ability of emergency processing so as to repair the network and ensure its normal operation. Therefore, starting from the protection of critical infrastructure network, further research should focus on how to improve the utilization of limited emergency resources and resist the propagation of cascading failure in the whole network.
